# Dominance of the coral *Pocillopora acuta* around Phuket Island in the Andaman Sea, Thailand

**DOI:** 10.1002/ece3.10724

**Published:** 2023-11-13

**Authors:** Anna Fiesinger, Christoph Held, Andrea L. Schmidt, Lalita Putchim, Frank Melzner, Marlene Wall

**Affiliations:** ^1^ GEOMAR Helmholtz Centre for Ocean Research Kiel Kiel Germany; ^2^ Department of Biology University of Konstanz Konstanz Germany; ^3^ Alfred‐Wegener‐Institut Helmholtz‐Zentrum für Polar‐ und Meeresforschung Bremerhaven Germany; ^4^ Cooperative Institute for Marine and Atmospheric Research University of Hawai‘i at Manoa Honolulu Honolulu USA; ^5^ Phuket Marine Biological Centre Phuket Thailand

**Keywords:** biodiversity, haplotypes, Indian Ocean, *Pocillopora*, species delimitation, species diversity

## Abstract

*Pocillopora damicornis* (Linnaeus, 1758), a species complex, consists of several genetic lineages, some of which likely represent reproductively isolated species, including the species *Pocillopora acuta* Lamarck, 1816. *Pocillopora acuta* can exhibit similar morphological characteristics as *P. damicornis*, thus making it difficult to identify species‐level taxonomic units. To determine whether the *P. damicornis*‐like colonies on the reefs in the Andaman Sea (previously often identified as *P. damicornis*) consist of different species, we sampled individual colonies at five sites along a 50 km coastal stretch at Phuket Island and four island sites towards Krabi Province, Thailand. We sequenced 210 coral samples for the mitochondrial open reading frame and identified six distinct haplotypes, all belonging to *P. acuta* according to the literature. Recently, *P. acuta* was observed to efficiently recolonize heat‐damaged reefs in Thailand as well as globally, making it a potentially important coral species in future reefs. Specifically in the light of global change, this study underscores the importance of high‐resolution molecular species recognition, since taxonomic units are important factors for population genetic studies, and the latter are crucial for management and conservation efforts.

## Graphical abstract

*Pocillopora damicornis* (Linnaeus, 1758), a species complex, consists of several genetic lineages, some of which likely represent reproductively isolated species, including the species *Pocillopora acuta* Lamarck, 1816. *P. acuta* can exhibit similar morphological characteristics as *P. damicornis*, thus making it difficult to identify species‐level taxonomic units. To determine whether the *P. damicornis*‐like colonies on the reefs in the Andaman Sea (previously often identified as *P. damicornis*) consist of different species, we sampled individual colonies at five sites along a 50 km coastal stretch at Phuket Island and four island sites towards Krabi Province, Thailand. We sequenced 210 coral samples for the mitochondrial open reading frame and identified six distinct haplotypes, all belonging to *P. acuta* according to the literature.

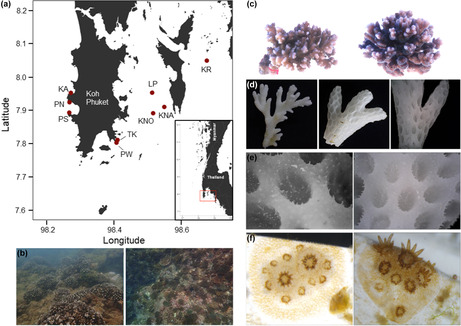

## 1 INTRODUCTION

*Pocillopora*, a widespread coral genus (Veron, 2000), harbors many fast‐growing reef‐builders that support a diverse and abundant community of other reef organisms such as echinoderms and crustaceans (Austin et al., 1980; López‐Pérez et al., 2017). *Pocillopora damicornis* (Linnaeus, 1758), previously identified solely based on its morphology (D'Croz & Maté, 2004; Mayfield et al., 2013, 2018), is known to be morphologically highly plastic (Schmidt‐Roach et al., 2013; Veron & Pichon, 1976). Phenotypic plasticity allows clonemates of *P. damicornis* genotypes to exhibit different morphologies based on environmental cues (Todd, 2008). This has called into question the ability to accurately identify species based solely on morphology (Flot et al., 2010; Pinzón & Lajeunesse, 2011; Schmidt‐Roach et al., 2012; Souter, 2010). Several taxa have been synonymized within *P. damicornis* and subsequently, grouped as *P. damicornis*‐like colonies (Pinzón et al., 2013) or the *P. damicornis* species complex (Schmidt‐Roach et al., 2013). Molecular evidence confirmed the expectations of several taxonomic species within the species complex (Schmidt‐Roach et al., 2013) and delineation of species boundaries has been the focus of many publications (e.g., Flot et al., 2010; Forsman et al., 2013; Johnston et al., 2017; Marti‐Puig et al., 2014; Oury et al., 2023; Pinzón & Lajeunesse, 2011; Schmidt‐Roach et al., 2014). Resolving species taxonomy is crucial, as different species have different functional roles in complex coral reef ecosystems (Boulay et al., 2014) and may respond differently to future changes. To distinguish species, a pocilloporid‐specific mitochondrial open reading frame (mtORF), which encodes a transmembrane protein (TMP362; Banguera‐Hinestroza et al., 2019), has been shown to be very informative (Flot et al., 2008; Flot & Tillier, 2007; Johnston et al., 2018).

Studies focusing on the Andaman Sea suggested that *P. damicornis*‐like corals belong to the *P. damicornis* type *β* or type *γ* lineages (Pinzón et al., 2013; Schmidt‐Roach et al., 2013) that have now been assigned species status as *P. acuta* Lamarck, 1816 and *P. verrucosa* Ellis & Solander, 1786, respectively (Schmidt‐Roach et al., 2014). *Pocillopora damicornis* type *β* (sensu Schmidt‐Roach et al., 2013) corresponds to mtORF Type 5 (sensu Pinzón et al., 2013), Type F (sensu Souter et al., 2009), Type b (sensu Flot et al., 2008) as well as ORF18 and ORF19 found within the Primary Species Hypothesis PSH05 proposed by Gélin, Postaire, et al. (2017). *Pocillopora damicornis* type *γ* (sensu Schmidt‐Roach et al., 2013) corresponds to mtORF Type 3 (sensu Pinzón et al., 2013), Type NF (sensu Souter et al., 2009), as well as ORF 35–48 within the Primary Species Hypothesis PSH13 proposed by Gélin, Postaire, et al. (2017). In the Andaman Sea, several authors refer to the prevalent species as *P. damicornis* (Brown et al., 2019; Kuanui et al., 2008; Phongsuwan & Chansang, 2012; Putchim et al., 2017) whereas others classify it as *P. acuta* (Rinkevich et al., 2016; Ying et al., 2021).

**FIGURE 1:**
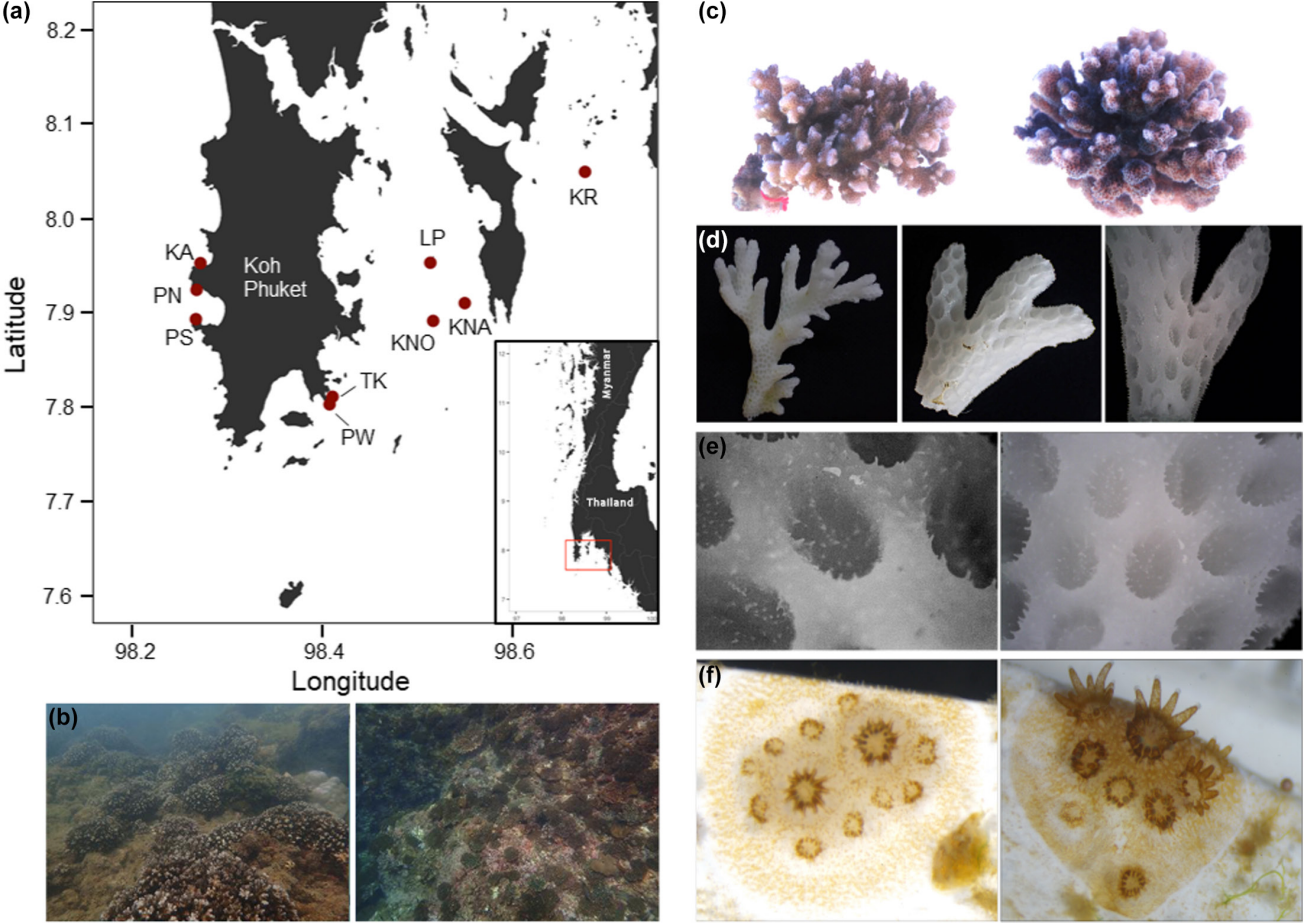
Map of the sampling sites of *Pocillopora acuta* around Phuket Island, Thailand. Site codes are: *KA* Kamala, *KNA* Khai Nai, *KNO* Khai Nok, *KR* Koh Nung Krabi, *LP* Le Pi, *PN* Patong North, *PS* Patong South, *PW* Panwa and *TK* Tang Khem. *Pocillopora* species were collected for this study from different reefs in the Thai Andaman Sea (a), where in‐situ *Pocillopora* can be quite dominant (b). Their branches are elongated with “acute” tips (c–d) and their skeletal calyx is oval as observed for both *P. damicornis* and *P. acuta* (e; Schmidt‐Roach et al., 2014). Coral recruits are formed from brooded larvae (collected from TK, PW) and colonies can fuse at early stage forming (f). Maps (a) were created with publicly available data from GADM.org.

Macromorphological and life‐cycle attributes, albeit insufficiently delimiting species within pocilloporids, suggest that *Pocillopora* colonies around Phuket Island are in fact *P. acuta*. These characters include fine and prolonged branches with “acute” branch tips, darker pigmentation surrounding the oral opening of the polyps, a brooding reproductive mode, as well as monthly planulation during or after the new moon (Kuanui et al., 2008; Schmidt‐Roach et al., 2012, 2014; Smith et al., 2019; Torres et al., 2020; Figure 1c,f).

Recent studies have highlighted the increasing dominance of pocilloporid corals in coral reef ecosystems (e.g., Poquita‐Du et al., 2019), particularly after massive disturbance events like heat waves (Edmunds et al., 2019), making them at least temporary winners of climate change (Laufkötter et al., 2020) and key conservation targets due to their rapid recolonization ability. To confirm, whether *P. acuta* is the dominant pocilloporid around Phuket Island, Thailand, the specific mtORF was amplified for 210 coral samples collected at nine sites. Such taxonomic data is critical for assessing the distribution of this important species and for effective coral reef management and conservation efforts in light of climatic change.

## 2 MATERIALS AND METHODS

### 2.1 Sampling sites and procedure

Coral samples were taken at nine different sites in the Andaman Sea around Phuket Island, Thailand (Figure 1) located in the Northeastern Indian Ocean, in the Eastern Bay of Bengal. The sampling locations comprise the western edge of Phuket Island (Kamala (KA), Patong North (PN) and Patong South (PS)), four islands towards Krabi Province (Khai Nai (KNA), Khai Nok (KNO), Ko Nung Krabi (KR) and Le Pi (LP)) and two sites around the Panwa peninsula (Panwa (PW) and Tang Khem (TK)). The two sites PW and TK are situated only 0.7 km apart, whereas a larger distance exists between both PW and TK and the sites on the West (18–23 km) as well as the islands towards Krabi Province (15–35 km). For each colony of *P. acuta* sampled, a small branch was clipped and stored in seawater until further processing in the lab at the Phuket Marine Biological Centre (PMBC, Phuket, Thailand). Samples were fixed in a solution of dimethyl sulfoxide, disodium EDTA, and saturated NaCl (DESS buffer; Yoder et al., 2006) and transferred to the lab facilities at GEOMAR Helmholtz Centre for Ocean Research Kiel, for molecular analyses. In total, 210 corals (5–65 per site) were analyzed for a hypervariable mitochondrial open reading frame (mtORF; Flot & Tillier, 2007).

### 2.2 Molecular work and data analysis

**FIGURE 2:**
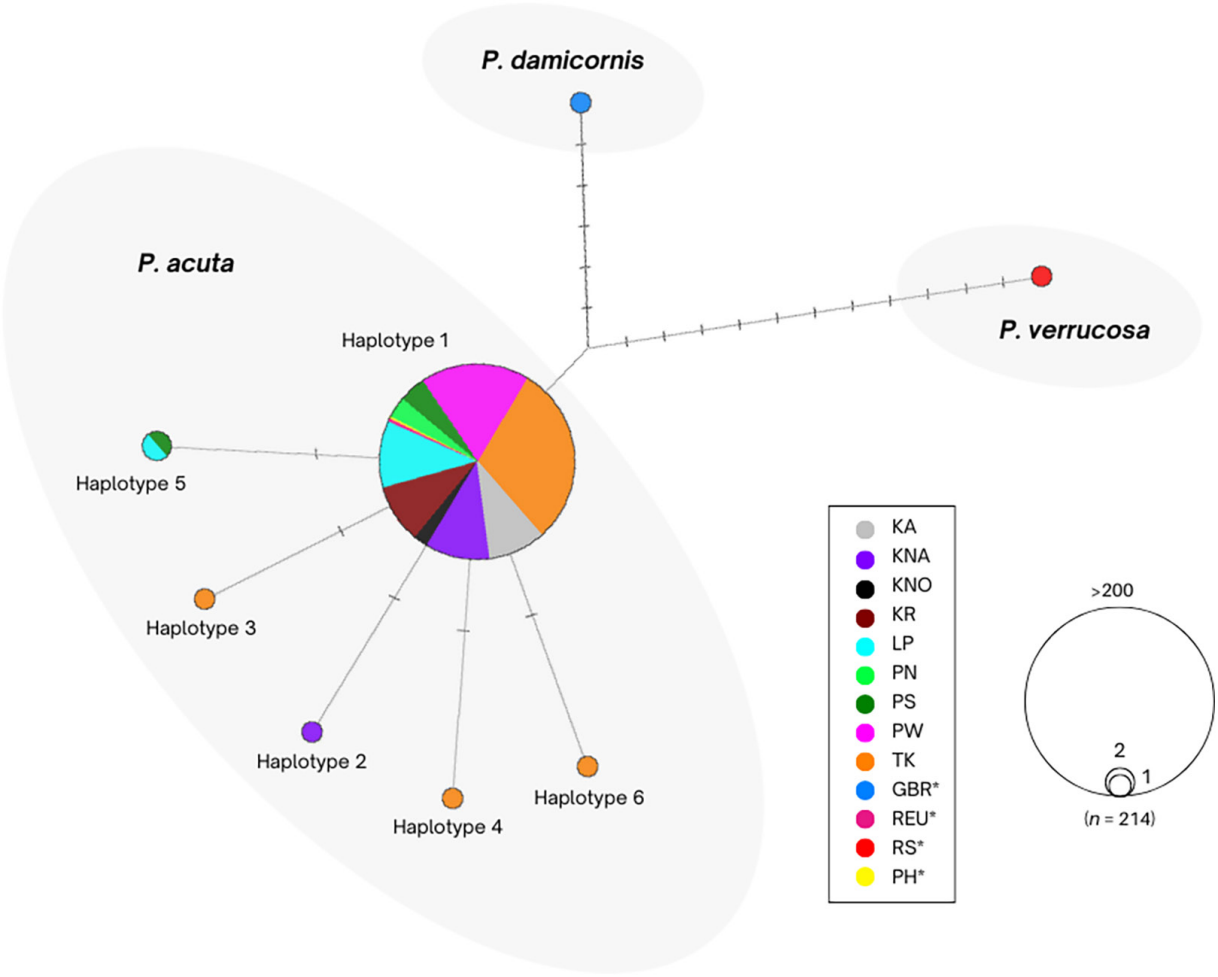
Minimum‐spanning haplotype network of mtORF DNA sequences from nine sampling sites around Phuket Island, Thailand. The alignment consisted of 210 sequences, 828 bp length, from several distinct reefs (*KA* Kamala, *KNA* Khai Nai, *KNO* Khai Nok, *KR* Koh Nung Krabi, *LP* Le Pi, *PN* Patong North, *PS* Patong South, *PW* Panwa and *TK* Tang Khem; see Figure 1). Sequences were referenced against two distinct *P. acuta* haplotypes found in the Philippine archipelago (PH) by Torres and Ravago‐Gotanco (2018) and on La Réunion (REU) by Gélin, Fauvelot, et al. (2017). *P. verrucosa* and *P. damicornis* references are from the Red Sea (RS) identified by Robitzch et al. (2015) and from the Great Barrier Reef (GBR) identified by Schmidt‐Roach et al. (2013), respectively. *Sequences obtained from GenBank.

DNA was extracted from coral tissue samples using an Ethanol precipitation protocol (Baker & Cunning, 2016). DNA quantity and quality from each sample was assessed with a NanoDrop® 2000 spectrophotometer and the quality was further screened from a subsample by gel electrophoresis. An mtORF was amplified using primers developed and described by Flot and Tillier (2007) for corals of the genus *Pocillopora* (FATP6.1 5′‐TTTGGGSATTCGTTTAGCAG‐3′ and RORF 5′‐SCCAATATGTTAAACASCATGTCA‐3′) for determination of mitochondrial haplotype lineages. PCR reactions were conducted as described in Robitzch et al. (2015): 30 μL reactions contained 15 μL Qiagen HotStarTaq *Plus* Master Mix, 1.88 μL primers (each primer at 20 μM, final concentration 1.25 μM), 8.74 μL NFW and 2.5 μL template DNA (10–40 ng/μL). Cycle conditions were: 95°C for 5 min + 35 × [94°C for 30 s, 58°C for 45 s, 72°C for 60 s] + 72°C for 10 min. Gel pictures were taken on 1% agarose gels with 1 kb GeneRuler DNA ladder (Thermo Scientific) as a ladder for all samples. Finally, 25 μL of each PCR product was sent to the Institute of Clinical Molecular Biology in Kiel (IKMB) for PCR cleanup and bidirectional Sanger sequencing on an ABI 3730xl DNA Analyzer. For the analyses, the respective forward and reverse sequences were assembled, aligned, and trimmed in CodonCode Aligner v9.0.1.3 (CodonCode Corporation). The contigs were checked for gaps and insertion/deletion polymorphisms (indels). Due to low quality, 26 sequences could not be assembled into contigs; therefore, they were excluded from the dataset after comparing readable positions to the unambiguous sequences. Sequences were identified using a BLAST search against the blastn database using the NCBI website (https://blast.ncbi.nlm.nih.gov/Blast.cgi). The resulting alignment of unambiguous, high‐quality reads was converted in MEGA X (Stecher et al., 2020) for further analysis of distinct haplotypes in DNAsp v6 (Rozas et al., 2017). Thereafter, the resulting NEXUS file was exported into Network 10.2.0.0 (https://fluxus‐engineering.com) to build a median‐joining network (Bandelt et al., 1999). Therefore, sequences of the sister species *P. verrucosa* from the Indo‐Pacific (GenBank accession number: KP238128) and *P. damicornis* from the Great Barrier Reef (GenBank accession number: JX625077) were obtained from GenBank (Figure 2; Pinzón et al., 2013; Schmidt‐Roach et al., 2013).

## 3 RESULTS AND DISCUSSION

Corals of the genus *Pocillopora* exhibit a high phenotypic plasticity, fostering the application of molecular techniques such as markers targeting mtORF or internal transcribed spacer (ITS) regions (Flot et al., 2008; Flot & Tillier, 2007; Marti‐Puig et al., 2014) for species identification. Furthermore, concepts like the Primary Species Hypothesis (PSH), which relies on morphological discriminations and/or molecular markers coupled with criteria such as biological or ecological considerations (Pante et al., 2015), can lead to the possible delineation of new species. In this study, 210 *P. acuta* colonies were sampled and a fragment of the mtORF (828 bp; GenBank accession numbers: MZ465117–MZ465326) was sequenced to identify the dominant species within the *P. damicornis* species complex around Phuket Island, Thailand. Unambiguous, high‐quality alignments were identified as *P. acuta* and compared to GenBank sequences of *P. damicornis* type *β*, now *P. acuta*, from La Réunion (accession numbers: KT879932–KT880039) and *P. acuta* from the Philippines (accession numbers: MH064356–MH064390). This dataset provided six distinct haplotypes (Figure 2). A total of 210 colonies yielded 204 samples corresponding to haplotype 1, which corresponds to one of four *P. acuta* haplotypes that were described in the Philippine archipelago by Torres and Ravago‐Gotanco (2018; GenBank accession number: MH064356) and ORF18 found in La Réunion (sensu Gélin, Fauvelot, et al., 2017; GenBank accession number: KT879932). Two of 210 samples corresponded to haplotype 5 and one sample each coincided with haplotypes 2, 3, 4, and 6. Interestingly, haplotypes 2–6 are novel haplotypes not yet found on other Andaman Sea reefs. However, this may be due to the lack of molecular data for *Pocillopora* corals in the region rather than the absence of corresponding haplotypes. For instance, the same haplotypes of *P. damicornis* type *α* and *β* could be found on either side of the Australian continent at Ningaloo Reef sites (Western Australia) and on the Great Barrier Reef (Eastern Australia; Thomas et al., 2014). On the contrary, Noreen et al. (2015) found distinct *Pocillopora* haplotypes at higher latitude subtropical and lower latitude tropical reefs on the Great Barrier Reef, despite prevalent oceanographic exchange via the Eastern Australian Current towards the subtropics. Only within a transition zone, both tropical and subtropical haplotypes coexisted.

Accurate species identification is critical for the widely distributed and researched genus *Pocillopora* to identify cryptic species and endemism since these have direct implications for conservation policies. Furthermore, the delimitation of taxonomic units for population genetic studies is crucial to avoid inaccurate estimations of genetic divergence and connectivity (Pante et al., 2015), which are the foundation of management and conservation programs. Here, of particular importance is the effect of reproduction on clonal structure, on larval dispersal potential and, therefore, on population connectivity. Ecological studies benefit from the ability to identify species, which allows for the characterization of significant differences and temporal variability in molecular physiological profiles between species (e.g., *P. damicornis* and *P. acuta*), with implications for resilience under long‐term stress (Mayfield et al., 2018). Furthermore, the inability to accurately characterize species as well as genetic clusters within species greatly reduces the ability to detect a response of the biota at various levels of hierarchy to ongoing climate change and undermines efforts guiding conservation efforts.

The findings within this study demonstrated the widespread occurrence of *Pocillopora acuta* around the island of Phuket, Thailand. Previous studies documented the existence of *P. acuta* in the Gulf of Thailand (Pinzón et al., 2013), the Indian Ocean (Gélin et al., 2018; Gélin, Fauvelot, et al., 2017) as well as the South China Sea (Poquita‐Du et al., 2017). Other studies have recently documented the dominance of this species on many coral reefs globally: A study conducted in Singapore evidenced that out of five pocilloporid species recorded since 1838, only colonies of *P. acuta* are remaining to date (Poquita‐Du et al., 2019). Since at least the 1970s, Mo'orea has experienced substantial reduction in coral cover, resulting in a predominant recovery of corals of the genus *Pocillopora* following each disturbance (Adjeroud et al., 2018; Berumen & Pratchett, 2006; Holbrook et al., 2018; McWilliam et al., 2020). A mass bleaching event in 2010 resulted in 100% reduction of coral cover (Edmunds et al., 2019); however, a high density of sexually reproduced offspring of *Pocillopora* colonies accelerated coral community recovery, followed by the recovery of other coral taxa. The impact of the 2010 mass bleaching event was heterogeneous across ocean basins with the Indian Ocean and Australasia more severely affected than the Pacific Ocean (Alemu & Clement, 2014; Guest et al., 2012; Hoeksema et al., 2012; Sutthacheep et al., 2013). Overall coral cover decline of up to 90% occurred in Southeast Asia, specifically in reefs around Indonesia, Malaysia, and Thailand (Tun et al., 2010). Around Phuket Island, specifically at the site TK, coral species diversity was high until 2007 and the genus *Pocillopora* comprised only a minor fraction (5% of total coral cover; Brown et al., 2019). A follow‐up survey in 2016 found a major shift in species composition, with *Pocillopora* colonies dominating (55% of total coral cover; Brown et al., 2019). Furthermore, fast‐growing corals like *Acropora* and *Pocillopora* were severely harmed in the bleaching event in 2010 but coped well with thermal stress during the subsequent bleaching episode in 2016 (Putchim et al., 2017). As a result of the 2010 bleaching event, adapted genotypes might have recolonized reefs around Phuket Island, therefore establishing the dominance of *Pocillopora acuta* colonies in the area (Fiesinger et al., 2023).

Since the genus *Pocillopora* comprises many species (Pinzón et al., 2013), it is likely that not all are able to withstand the ongoing and escalating climatic perturbations predicted for the upcoming decades. However, the species and more specifically the genotypes that do survive might be more suitable and thus, selected for future warming conditions (Hume et al., 2022). By identifying the highly connected and more stress‐tolerant species and genotypes within the genus, such as *P. acuta*, targeted management efforts may facilitate rapid recolonization of future reefs thereby catalyzing their recovery (Hume et al., 2022). Because many ecosystems are naturally prone to pulse disturbances, “regeneration taxa,” which are able to rapidly recolonize an area after severe disturbance events, are critical (McWilliam et al., 2020). However, a shift away from long‐lived taxa to rapid recolonizers alters the functional trait assemblage of coral reef ecosystems (Lavorel et al., 1997; McWilliam et al., 2020). In this context, it is necessary to point out that *P. acuta* has been shown to express special properties: intracolonial genetic variability such as mosaicism and chimerism (Oury et al., 2020; Rinkevich et al., 2016) as well as polyploidy (Stephens et al., 2023). While coral chimerism may enhance within‐colony biodiversity that may counter the detrimental effects of changing ecosystems on genetic and phenotypic diversity (Rinkevich, 2019), triploidy may under certain circumstances outcompete diploid genotypes (Stephens et al., 2023) through beneficial traits as observed in aquaculture (e.g., increased growth, pathogen resistance; Guo & Allen, 1994; Zhou & Gui, 2017) but may come at the cost of infertility (as observed in other invertebrates; Kang et al., 2013). Therefore, the dominance of a single coral species together with its peculiar characteristics may have implications for the functioning of Andaman Sea reefs that need to be the focus of future studies.

In conclusion, the coral reefs around Phuket Island are dominated by *P. acuta* colonies and it is likely that recent monitoring efforts have identified these as *P. damicornis*. However, it cannot be discounted that in the past, different species within the *P. damicornis* species complex possibly coexisted that have since been outcompeted by fast‐growing *P. acuta* colonies after massive bleaching events. It would be desirable to expand the sampling area and include more offshore sites that potentially comprise a higher pocilloporid diversity. Ancient DNA from museum samples or coral skeletons could provide an overview of which *Pocillopora* species were present in the area in the past. We should treat species delimitation carefully as it can be challenging and shift in a changing ocean. A more integrative approach should be followed in the future by combining molecular markers with additional physiological and biological criteria assessments to build up evolutionary species units. Such identifications are key for coral reef management and local as well as regional restoration efforts and can facilitate the identification of those genotypes that are most likely to withstand future climate conditions.

## AUTHOR CONTRIBUTIONS

**Anna Fiesinger:** Conceptualization (equal); data curation (lead); formal analysis (lead); investigation (equal); methodology (lead); validation (lead); visualization (lead); writing – original draft (lead); writing – review and editing (lead). **Christoph Held:** Formal analysis (equal); investigation (equal); methodology (equal); software (equal); supervision (equal); validation (equal); writing – original draft (supporting); writing – review and editing (supporting). **Andrea L. Schmidt:** Data curation (supporting); resources (supporting); writing – review and editing (supporting). **Lalita Putchim:** Data curation (equal); funding acquisition (supporting); project administration (supporting); resources (equal); writing – review and editing (equal). **Frank Melzner:** Funding acquisition (lead); investigation (equal); project administration (equal); supervision (equal); validation (equal); writing – original draft (equal); writing – review and editing (equal). **Marlene Wall:** Conceptualization (lead); data curation (supporting); funding acquisition (lead); investigation (lead); methodology (supporting); project administration (lead); supervision (lead); validation (equal); visualization (equal); writing – original draft (equal); writing – review and editing (equal).

## CONFLICT OF INTEREST STATEMENT

The authors declare no competing interests.

## Data Availability

The sequences obtained for the coral colonies analyzed in this study can be obtained from GenBank with the accession numbers MZ465117–MZ465326.
